# EFFICACY AND SAFETY OF ONE ANASTOMOSIS GASTRIC BYPASS IN SURGICAL TREATMENT OF OBESITY: SYSTEMATIC REVIEW AND META-ANALYSIS OF RANDOMIZED CONTROLLED TRIALS

**DOI:** 10.1590/0102-6720202400021e1814

**Published:** 2024-08-30

**Authors:** Tiago Rafael ONZI, Wilson SALGADO, Eduardo Lemos de Souza BASTOS, Anna Carolina Batista DANTAS, Lyz Bezerra SILVA, Alvaro Albano de OLIVEIRA, Luca Schiliró TRISTÃO, Clara Lucato dos SANTOS, Wanderley Marques BERNARDO, Matheus Pedrotti CHAVEZ

**Affiliations:** 1 Universidade Federal de Santa Catarina, General and Digestive Surgery Service – Florianópolis (SC), Brazil;; 2 Universidade de São Paulo, Department of Surgery and Anatomy – Ribeirão Preto (SP), Brazil;; 3 Faculdade de Medicina de Marília, Department of Gastrointestinal Surgery – Marília (SP), Brazil;; 4 Universidade de São Paulo, Bariatric and Metabolic Surgery Unit – São Paulo (SP), Brazil;; 5 University College London Hospital NHS Foundation Trust, Department of Bariatric and Metabolic Surgery, London – United Kingdom;; 6 Santa Casa de Misericórdia de Itabuna, Bariatric and Metabolic Service – Salvador (BA), Brazil;; 7 Lusíada Centro Universitário, Department of Evidence Based Medicine – Santos (SP), Brazil;; 8 Universidade de São Paulo, Faculty of Medicine, Department of Evidence Based Medicine – São Paulo (SP), Brazil;; 9 Universidade Federal de Santa Catarina, Faculty of Medicine – Florianópolis (SC), Brazil.

**Keywords:** Gastric Bypass, Gastrectomy, Bariatric Surgery, Obesity, Derivação Gástrica, Gastrectomia, Cirurgia Bariátrica, Obesidade

## Abstract

**BACKGROUND:**

One anastomosis gastric bypass (OAGB) has gained prominence in the search for better results in bariatric surgery. However, its efficacy and safety compared to Roux-en-Y gastric bypass (RYGB) and sleeve gastrectomy (SG) remain ill-defined.

**AIMS:**

To compare the efficacy and safety of OAGB relative to RYGB and SG in the treatment of obesity.

**METHODS:**

We systematically searched PubMed, EMBASE, Cochrane Library, Lilacs, and Google Scholar databases for randomized controlled trials comparing OAGB with RYGB or SG in the surgical approach to obesity. We pooled outcomes for body mass index, percentage of excess weight loss, type-2 diabetes mellitus remission, complications, and gastroesophageal reflux disease. Statistical analyses were performed with R software (version 4.2.3).

**RESULTS:**

Data on 854 patients were extracted from 11 randomized controlled trials, of which 422 (49.4%) were submitted to OAGB with mean follow-up ranging from six months to five years. The meta-analysis revealed a significantly higher percentage of excess weight loss at 1-year follow-up and a significantly lower body mass index at 5-year follow-up in OAGB patients. Conversely, rates of type-2 diabetes mellitus remission, complications, and gastroesophageal reflux disease were not significantly different between groups. The overall quality of evidence was considered very low.

**CONCLUSIONS:**

Our results corroborate the comparable efficacy of OAGB in relation to RYGB and SG in the treatment of obesity, maintaining no significant differences in type-2 diabetes mellitus remission, complications, and gastroesophageal reflux disease rates.

## RESUMO

**RACIONAL:**

O bypass gástrico de uma anastomose (BGU tem ganhado destaque na busca por melhores resultados na cirurgia bariátrica. No entanto, a eficácia e segurança do BGUA, em relação ao bypass gástrico em Y de Roux (BGYR) e à gastrectomia vertical (GV), permanecem imprecisas.

**OBJETIVOS:**

Comparar a eficácia e segurança do BGUA em relação ao BGYR e GV no tratamento da obesidade.

**MÉTODOS:**

Realizamos uma busca sistemática nas bases de dados do PubMed, EMBASE, Cochrane, Lilacs e Google Scholar por ensaios clínicos randomizados que comparassem o BGUA ao BGYR ou GV na abordagem cirúrgica da obesidade. Agrupamos resultados para índice de massa corporal, porcentagem da perda de peso em excesso, remissão do diabetes mellitus tipo 2, complicações e taxas de doença do refluxo gastroesofágico. As análises estatísticas foram realizadas empregando o software R, versão 4.2.3.

**RESULTADOS:**

Dados de 854 pacientes foram extraídos de 11 ensaios clínicos randomizados, dos quais 422 (49,4%) foram submetidos ao BGUA, com seguimento médio variando de seis meses a cinco anos. Na análise combinada, o BGUA esteve associado a uma porcentagem da perda de peso em excesso significativamente maior no acompanhamento de um ano e a um índice de massa corporal significativamente menor no acompanhamento de cinco anos em pacientes submetidos ao BGUA. Por outro lado, as taxas de remissão da diabetes mellitus tipo 2, de complicações e da doença do refluxo gastroesofágico não foram significativamente diferentes entre os grupos. A qualidade geral das evidências foi considerada muito baixa.

**CONCLUSÕES:**

Nossos resultados corroboram a eficácia comparável do BGUA em relação ao BGYR e GV no tratamento da obesidade, mantendo diferenças não significativas nas taxas de remissão da diabetes mellitus tipo 2, complicações e doença do refluxo gastroesofágico.

## INTRODUCTION

Obesity is a growing condition worldwide, both in underdeveloped and developing countries^17^. Despite increasing global efforts to reduce the growth rate, a recent publication by the World Obesity Federation shows a projection that more than 50% of the world’s population will be overweight or obese by 2035^58^.

Although the existence of significant and promising new drugs in the approach to obesity disease^33^, bariatric and metabolic surgery remains the most effective and durable treatment^55^. Roux-en-Y gastric bypass (RYGB) surgery was the dominant procedure for many years^16,56,57^ but has been surpassed by sleeve gastrectomy (SG) in recent years^6^.

Different surgical techniques have been gaining ground in the search for better results with fewer complications^44^. The one anastomosis gastric bypass (OAGB), first introduced by Dr. Rutledge in 1997, has been upheld by several publications with encouraging results^11,43,45^. OAGB is a “combined procedure” that incorporates both a “restrictive” and a “hypoabsorptive” component^41,51,56^. Demonstrating remarkable efficacy in mitigating obesity-related comorbidities, it also offers a good quality of life while maintaining a manageable complication rate^13,28,29,56^.

The escalating prevalence of OAGB in Europe and the Asia-Pacific has elevated its status to the third most commonly performed bariatric surgery, ranking behind SG and RYGB^4,5,13^.

Recent randomized controlled trials (RCTs) have compared OAGB with the main bariatric surgeries, SG and RYGB, showing promising results^15,21,31,42,44,50^. However, these studies included different populations with variations in body mass index (BMI) and surgical techniques, displaying divergent outcomes. Therefore, we performed a comprehensive systematic review and meta-analysis of all published RCTs, aiming at providing pooled effect estimates regarding the efficacy and safety of OAGB in the treatment of obesity, as compared with SG and RYGB.

## METHODS

This systematic review and meta-analysis followed the Preferred Reporting Items for Systematic Reviews and Meta-Analysis (PRISMA) guidelines, including design, implementation of steps, analysis, and description of results^39^.

### Search strategy

The databases PubMed, Embase, Cochrane Library, Latin American and Caribbean Health Sciences Literature (Lilacs), and Google Scholar were systematically searched from inception to May 2023 with the following search strategy: (Bariatrics OR Bariatric Surgery OR Bariatric Surgical Procedures OR Bariatric Surgical Procedure OR Bariatric Surgeries OR Gastric Bypass) AND (One Anastomosis Gastric Bypass OR OAGB). Aiming at the inclusion of additional studies, the references of the included articles and systematic reviews of the literature were evaluated.

### Inclusion criteria

Studies with the following criteria were included:

1. RCTs
2. Comparing OAGB with RYGB or SG and
3. Reporting at least one of the outcomes of interest.

Incomplete or unpublished trials, non-RCTs, and conference abstracts were excluded. There were no restrictions on language or publication date.

### Data extraction

Two authors (L.C.T. and C.L.S.) independently extracted baseline characteristics and data outcomes following predefined search criteria. Disagreements were resolved by consensus between the two investigators and the senior author (W.M.B.). Data presented in the studies from the longest follow-up analysis with control group comparison were extracted for analyses. For data handling and conversion, the Cochrane Handbook for Systematic Reviews of Interventions guidelines were used^19^. The population of different publications from the same trial was only counted once.

### Outcomes

The primary outcomes were the change in BMI (kg/m^2^) compared to the baseline value at six months, one year, and five years, and the percentage of excess weight loss (%EWL) at one year and five years. Accordingly, secondary outcomes were type-2 diabetes mellitus (T2DM) remission, complications, and gastroesophageal reflux disease (GERD) rates at the longest follow-up.

The definition of T2DM remission was heterogeneous among the included studies. Therefore, data of this endpoint was collected as equally as reported by each RCT^19,31,43,48,50^, precluding data manipulation.

### Risk of bias and evidence quality

Two authors (T.O. and L.C.T.) independently assessed the risk of bias, and disagreements were resolved with the senior author (W.M.B.). The risk of bias assessment followed the recommendations of the Cochrane Handbook for Systematic Reviews of Interventions, with the Cochrane Collaboration’s tool for assessing the risk of bias in randomized trials (Rob-2)^52^. Each trial received a score of high, low, or some concerns risk of bias in five domains: randomization process; deviations from the intended interventions; missing outcomes; measurement of the outcome; and selection of reported results.

The evidence quality was assessed according to the Grading of Recommendation, Assessment, Development and Evaluation (GRADE) guidelines^36^. Very low, low, moderate, or high-quality evidence grades were designed for the outcomes based on the risk of bias, inconsistency of results, imprecision, publication bias, and magnitude of treatment effects.

### Statistical analysis

Endpoints were analyzed by weighted mean differences (WMDs) or risk ratios (RRs) with 95% confidence intervals (CIs) to compare treatment effects. Cochrane Q-test and I^2^ statistics were applied to assess heterogeneity; p<0.100 and I^2^>50% were considered significant^20^. DerSimonian and Laird random-effect models were used for all endpoints, including the surgery performed in the control group^14^. Statistical analyses were performed using R software, version 4.2.3 (R Core Team, 2021, Vienna, Austria).

## RESULTS

### Study selection and baseline characteristics

**Figure 1:**
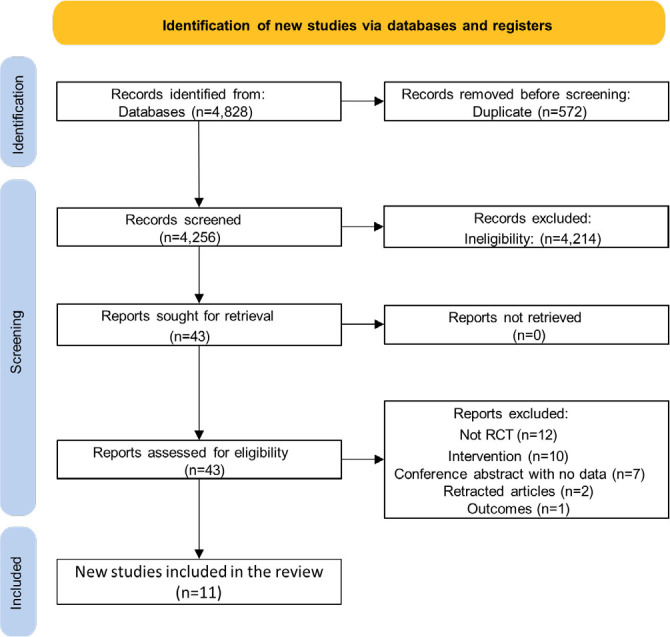
Preferred Reporting Items for Systematic Reviews and Meta-Analysis (PRISMA) flow diagram of study screening and selection.

The systematic search yielded 4,828 studies. After removing duplicates and ineligible studies by title or abstract, 43 articles were fully reviewed for inclusion and exclusion criteria. Of these, 11 were included in this meta-analysis^15,21,24,30,31,44,48-50^. Data from one RCT was reported in three different publications^21,48,50^. A total of 854 patients were assessed, of whom 422 (49.4%) were submitted to OAGB. At baseline, the mean age ranged from 31 to 46 years, the mean body weight from 109.5 to 137.5 kg, and the mean BMI from 42.7 to 49.9 kg/m^2^. A flow diagram describing the selection process (inclusion and exclusion) is shown in Figure 1, and the basic characteristics of the included studies are summarized in Table 1.

**Table 1:** Baseline characteristics of included studies.

Author	Follow-up (years)	Intervention	Control group	Patients	Sample size IG/CG	Initial BMI (kg/m^2^)	
						IG	CG
Lee et al.^30^	2	MGB/OAGB	RYGB	Morbid obesity	40/40	44.80±8.80	43.80±4.80
Seetharamaiah et al.^49^	1	OAGB	LSG	Obesity	101/100	44.32±7.88	44.57±7.16
Shivakumar et al.,^51^	3	OAGB	LSG	Obesity	101/100	44.32±7.88	44.57±7.16
Robert et al.^42^	2	OAGB	RYGB	Obesity	129/124	43.80±6.10	43.90±5.10
Kraljevic et al.^28^	1	LOAGB	LRYGB	Obesity	40/40	NA	NA
Eskandaros et al.^15^	1	LOAGB	LRYGB	GERD+obesity	40/40	49.78±3.40	50.01±3.50
Jain et al.^21^	5	OAGB	LSG	Obesity	73/71	45.32±8.24	44.89±7.94
Katayama et al.^24^	6 months	OAGB	RYGB	Obesity	10/10	43.20±3.70	43.10±3.90
Level et al.^31^	5	OAGB	RYGB	Obesity	9/24	42.90±5.50	42.60±5.90
Musella et al.^38^	1	MGB/OAGB	SG	GERD	28/30	48.50±8.90	47.50±7.30
Singh et al.^50^	4	LOAGB	LRYGB	Obesity+T2DM	25/24	47.00±6.70	44.70±4.90

IG: intervention group; CG: control group; BMI: body mass index; MGB: mini-gastric bypass; OAGB: one anastomosis gastric bypass; RYGB: Roux-en-Y gastric bypass; LSG: laparoscopic sleeve gastrectomy; LOAGB: laparoscopic one anastomosis gastric bypass; LRYGB: laparoscopic Roux-en-Y gastric bypass; GERD: gastroesophageal reflux disease; T2DM: type-2 diabetes mellitus; NA: not available; ± standard deviation; SG: sleeve gastrectomy.

### Body mass index

**Figure 2:**
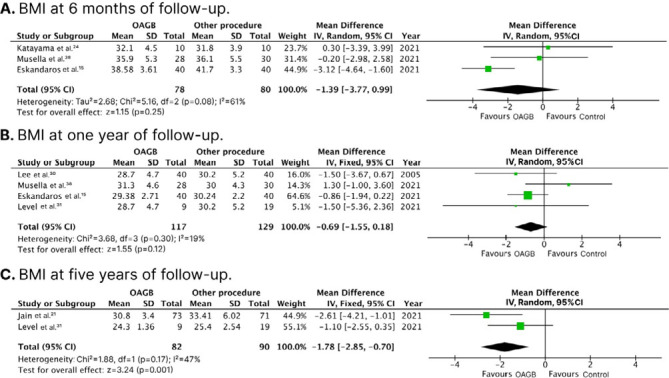
Forest plots of pooled comparisons of body mass index (kg/m^2^) endpoints. Figure 2-A: at 6-month follow-up; Figure 2-B: at 1-year follow up; Figure 2-C: at 5- year follow-up.

BMI: body mass index; CI: confidence interval; OAGB: one anastomosis gastric bypass; SD: standard deviation; df: degrees of freedom; p: p-value; I^2^: heterogeneity; z: standard normal distribution.

No significant differences were observed between groups in BMI at 6-month follow-up (WMD 1.39 kg/m^2^; 95%CI -3.77; 0.99; p=0.250; I^2^: 61%; Figure 2A) and 1-year follow-up (WMD -0.69 kg/m^2^; 95%CI -1.55; 0.18; p=0.120; I^2^: 19%; Figure 2B). However, at 5-year follow-up, OAGB was associated with a significant decrease in BMI compared with control (WMD -1.78 kg/m^2^; 95%CI -2.85; -0.70; p=0.001; I^2^: 47%; Figure 2C).

### Percentage of excess weight loss

**Figure 3:**
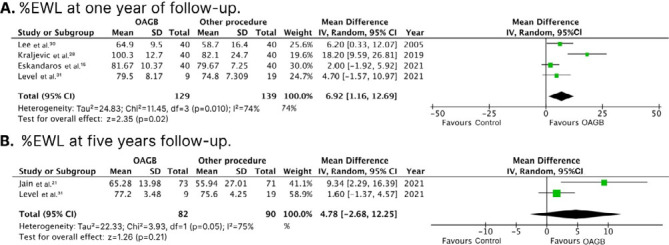
Forest plots of pooled comparisons of the percentage excess weight loss endpoints. Figure 3-A: at 1-year follow-up; Figure 3-B: at 5-year follow-up.

There was a significantly higher %EWL in the OAGB group at 1-year follow-up (WMD 6.92%; 95%CI 1.16; 12.69; p=0.020; I^2^: 74%; Figure 3A), compared with control. At 5-year follow-up, there was no significant difference between groups in %EWL (WMD 4.78%; 95%CI -2.68; 12.25; p=0.050; I^2^: 75%; Figure 3B).

%EWL: percentage of excess weight loss; CI: confidence interval; OAGB: one anastomosis gastric bypass; SD: standard deviation; df: degrees of freedom; p: p-value; I^2^: heterogeneity; z: standard normal distribution.

### Type-2 diabetes mellitus remission, complications, and gastroesophageal reflux disease rates

**Figure 4:**
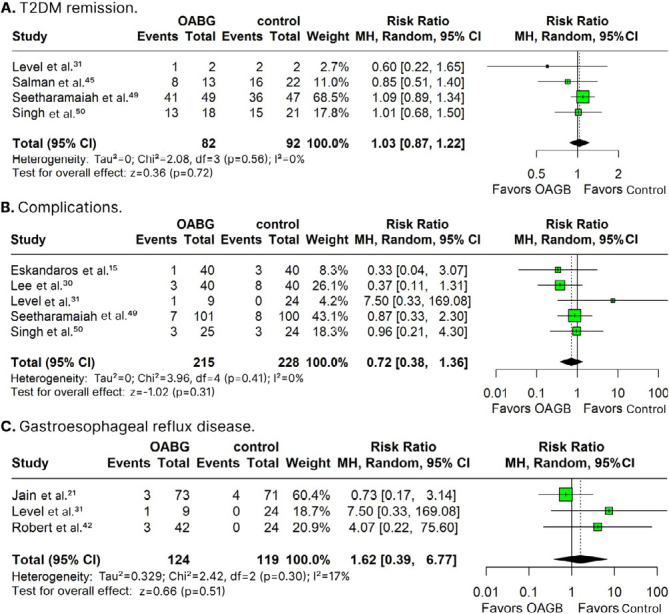
Forest plots of pooled comparisons of type-2 diabetes mellitus remission (Figure 4-A), complication rates (Figure 4-B) and GERD (Figure 4-A) endpoints.

T2DM: type-2 diabetes mellitus; CI confidence interval; OAGB: one anastomosis gastric bypass; SD: standard deviation; df: degrees of freedom; p: p-value; I^2^: heterogeneity; z: standard normal distribution; MH: Mantel-Haenszel random-effects model.

Patients submitted to OAGB showed similar rates of T2DM remission (RR 1.03; 95%CI 0.87; 1.22; p=0.720; I^2^: 0%; Figure 4A), complications (RR 0.72; 95%CI 0.38; 1.36; p=0.310; I^2^: 0%; Figure 4B), and GERD (RR 1.62; 95%CI 0.39; 6.77; p=0.510; I^2^: 17%; Figure 4C) in comparison with control.

Overall, among patients who underwent OAGB, there were three hemorrhages, two marginal ulcers, eight intraoperative complications, three early complications, and 22 late complications^16,17,18,23,29^. For those in the RYGB group, four intraoperative complications, five early complications, and 18 late complications were reported^30,31,42,50^. For patients who received SG, four hemorrhages and one anastomotic dehiscence were related^52^.

One study classified complications by the Clavien-Dindo score^42^. In the RYGB group, two cases (bowel obstruction and hemoperitoneum) were scored over grade 3 and required surgical management. In the OAGB group, one case (peritonitis) was scored over grade 3 and also required surgical treatment.

### Risk of bias and evidence quality

**Figure 5:**
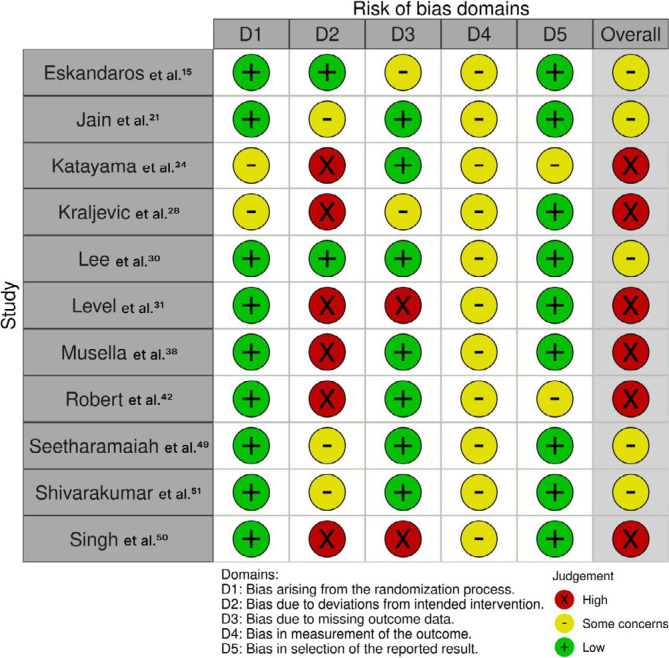
Critical appraisal of randomized controlled trials according to the Cochrane Collaboration’s tool for assessing risk of bias in randomized trials.

Figure 5 outlines individual assessments of each RCT included in this meta-analysis. Due to the assignment of some concerns or high risk of bias in one or more domains of the Cochrane Collaboration’s tool, five studies were deemed at some concerns risk of bias and six studies at high risk.

According to the GRADE assessment, BMI and %EWL outcomes were classified as very low-quality evidence (Table 2). The primary domains contributing to reduced evidence quality for these outcomes were inconsistency due to heterogeneity and imprecision, which resulted from a small number of RCTs assessing the outcome.

**Table 2:** Analysis of the quality of evidence (GRADE) in relation to the overall rate of occurrence of the assessed outcomes.

Certainty assessment								Patients (n)		Certainty of evidence
Outcome follow-up	Studies (n)	Study design	Risk of bias	Inconsistency	Indirectness	Imprecision	Others considerations	OAGB	Control	
BMI at 6-month	3	RCT	VS	Serious (I^2^>50)	NS	VS	None	78	80	⊕⭘⭕⭕ Very low
BMI at 1-year	4	RCT	VS	NS	NS	Serious	None	117	129	⊕⭘⭕⭕ Very low
BMI at 5-year	2	RCT	VS	NS	NS	Serious	None	82	90	⊕⭘⭕⭕ Very low
%EWL at 1-year	4	RCT	VS	Serious (I^2^>50)	NS	NS	None	129	139	⊕⭘⭕⭕ Very low
%EWL at 5-year	2	RCT	VS	Serious (I^2^>50)	NS	VS	None	82	90	⊕⭘⭕⭕ Very low

BMI: body mass index; OAGB: one anastomosis gastric bypass; RCT: randomized controlled trial; VS: very serious; NS: not serious; EWL: excess weight loss.

## DISCUSSION

In this systematic review and meta-analysis of eleven RCTs, comprising 854 patients, we compared OAGB to SG and RYGB for the treatment of obesity.

Our main findings were:

1. OAGB significantly decreased BMI at 5-year follow-up;
2. OAGB significantly improved %EWL at 1-year follow-up;
3. Non-significant differences were observed between OAGB and the control group regarding T2DM remission; and
4. OAGB and control groups exhibited comparable rates of complications and GERD at the longest follow-up.

For many years, RYGB was considered the gold standard procedure for obesity treatment^8,12^. However, other procedures have gained prominence in the pursuit of improved outcomes in bariatric surgery, with SG and OAGB being the most performed surgeries as an alternative to RYGB^25,54^. Meanwhile, despite OAGB being a less prevalent procedure, it has been associated with superior weight loss efficacy than the traditional RYGB approach due to the substantially longer biliopancreatic limb (BPL)^26,42,46^.

Many studies have been accomplished to enhance outcomes of bypass surgery by investigating limb lengths^9,10,22,40,41,47,53^. The length predominantly used in OAGB is 200 cm^35^. However, several studies advocate for a 150-cm BPL to minimize nutritional deficiencies while keeping an acceptable weight loss and comorbidities remission^1,9,27^. A recent meta-analysis comparing both 200 and 150 cm BPL in OAGB demonstrated that the 200 cm group achieved better weight loss outcomes and a comparable remission of comorbidities, at the expense of higher nutritional deficiency rates^6^. In our meta-analysis, the BPL in the included RCTs was mainly measured ranging from 180 to 220 cm.

Bariatric surgery is still the most efficacious and enduring intervention for severe obesity^5^. Nonetheless, 20 to 25% of patients experience weight regain after the surgical procedure, mainly due to a convergence of insufficient psychosocial counseling, high-calorie intake, and inadequate physical activity^1,3,7,18,23^. In this meta-analysis, OAGB was associated with a significantly higher %EWL and a significant decrease in BMI at 1- and 5-year follow-up, respectively. Accordingly, previous evidence comparing OAGB with RYGB exhibited increasing and significant %EWL values in individuals treated with OAGB after 1, 2, and 5 years of follow-up^34^. In light of these findings, it is essential to take into account the substantial long-term efficacy of OAGB, potentially contributing to decreased rates of weight regain after bariatric surgery.

Evidence-based knowledge has significantly expanded the management of obesity and associated comorbidities. Bariatric surgery has proven its efficacy for weight loss, expanding its action on T2DM remission^8,32^. In this study, the comparable effectiveness of OAGB with the control group showed a non-significant difference in T2DM remission. However, previous meta-analyses of RCTs comparing OAGB with RYGB found that OAGB delivered better remission rates for comorbidities^8^. Likewise, when compared with SG, previous meta-analyses have also supported the superiority of OAGB by increasing remission rates of T2DM^2,33^.

Other aspects should be considered when selecting the optimal bariatric surgical procedure, such as GERD^37,38^. Generally, OAGB is peformed with a wider gastric tube, leading to low intraluminal pressure and GERD^34^. In our pooled analysis, no significant difference in GERD rates was encountered between OAGB and the control group at follow-up. However, various publications indicated gastroesophageal reflux, typically bile reflux and its carcinogenic potential in the esophagus as prominent complications of OAGB^25^. Despite reports of these events in the literature being lower than expected, the International Federation for the Surgery of Obesity and Metabolic Disorders (IFSO) 2018 task force recommended that these complications remain a theoretical risk^13,35^. Therefore, as a point of discussion among experts, a recent consensus on patient selection for OAGB indicated that this treatment should not be offered to patients with grade C or D esophagitis or Barrett’s metaplasia^26^.

Overall, complication rates of surgical procedures appeared to be comparable. Likewise, in another meta-analysis by Ali et. al., OAGB and SG were also associated with non-significant differences in complication rates^2^. Additionally, in a direct comparison between OAGB and RYGB, other studies also found low and comparable rates of complications^8,34^. Due to the long-stapled lines and gastrointestinal anastomoses, the main risks associated with bariatric procedures are linked to the possibility of leaks or hemorrhages. Mangoulitis et al., in a previous meta-analysis, examined these endpoints and found comparable incidences between OAGB and RYGB, supporting their safety^34^.

This study has limitations. First, there was moderate to high heterogeneity in some outcomes analyzed, such as the %EWL. However, this effect might be inherited from the small sample size and the small number of studies that reported this endpoint. Second, the absence of patient-level data regarding BMI and %EWL outcomes, precisely for participants with T2DM, precluded a potential subgroup analysis. Third, although this meta-analysis accomplished a comprehensive comparison of OAGB with RYGB and SG, the RCTs analyzed had high or some concerns at risk of bias assessment, limiting definite conclusions. Additional high-quality RCTs are expected to shed further light on the OAGB efficacy and safety.

## CONCLUSIONS

Our meta-analysis of RCTs shows that OAGB surgery, compared with RYGB and SG, has significantly higher %EWL at 1-year follow-up and a significant decrease in BMI at 5-year follow-up, with a comparable rate of complications. Although bile reflux remains a theoretical risk after OAGB, our findings endorse OAGB as an effective and safe treatment for obese patients.
